# Hardness Prediction of Laser Powder Bed Fusion Product Based on Melt Pool Radiation Intensity

**DOI:** 10.3390/ma15134674

**Published:** 2022-07-03

**Authors:** Ting Zhang, Xin Zhou, Peiyu Zhang, Yucong Duan, Xing Cheng, Xuede Wang, Guoquan Ding

**Affiliations:** 1 Key Laboratory of Airborne Plasma Dynamics, Air Force Engineering University, Xi’an 710038, China; wxy_zhangting@163.com (T.Z.); peiyuzhang1128@gmail.com (P.Z.); 22920152203689@stu.xmu.edu.cn (Y.D.); chengxing0520@hotmail.com (X.C.); 18092623195@163.com (X.W.); ding072873@163.com (G.D.); 2 PLA Unit 93119, Jiuquan 735000, China; 3 Xi’an Aerospace Mechatronics & Intelligent Manufacturing Co., Ltd., Xi’an 710038, China

**Keywords:** laser powder bed fusion, melt pool radiation intensity, random forest, XGBoost, LightGBM

## Abstract

The quality stability and batch consistency of laser powder bed fusion products are key issues that must be solved in additive manufacturing. The melt pool radiation intensity data of laser powder bed fusion contain a significant amount of forming process information, and studies have shown that the analysis of melt pool radiation intensity using data-driven methods can achieve online quality judgment; however, there are still speed and accuracy problems. In this study, we propose a data-driven model for hardness predictions of laser powder bed fusion products based on process parameters fused with power spectrum features of melt pool intensity data, which quickly and accurately predicts the microhardness of laser powder bed fusion specimens and can make constructive guidance for closed-loop feedback quality regulation in practical production. The effects of three integrated learning models, Random Forest, XGBoost and LightGBM, are also compared. The results indicate that random forest has the highest prediction accuracy in this dataset; however, it has the limitation of slow training and prediction speeds. The LightGBM algorithm has the fastest training and prediction speeds, about 1.4% and 4.4% of the random forest, respectively; however, the prediction accuracy is lower than that of random forest and XGBoost. XGBoost has the best overall comparative performance with adequate training and prediction speeds, about 23.7% and 37.9% of the random forest, respectively, while ensuring a specified prediction accuracy, which is suitable for application in engineering practices.

## 1. Introduction

Laser powder bed fusion (LPBF) is a new additive manufacturing technology that uses a high-energy laser heat source to irradiate a pre-coated thin metal powder, melt it locally, and then solidify it by cooling and forming it layer-by-layer [1]. LPBF is capable of directly producing high-density, high-precision, and arbitrarily complex-shaped metal parts and has been used in a variety of industries, such as aerospace, automotive, biomedical, and tooling [2]. Despite the advantages of LPBF and its promising development, ensuring consistent and reproducible forming quality remains the biggest barrier to its industrial maturity [3].

Online monitoring and in situ quality evaluation of the LPBF process, and thus online quality control, is considered to be an effective method for solving product quality stability and reproducibility problems, where the morphology, size, and temperature of the melt pool significantly impact the quality of the formed parts. Therefore, in situ online monitoring of the melt pool is essential. Several studies have been conducted to achieve online monitoring of the melt pool in LPBF using different devices, including high-speed cameras, pyrometers, and photodiodes [4]. The use of photodiodes as sensors for melt pool signal acquisition has the advantages of low hardware cost, high monitoring sensitivity, good robustness, rapid response time, small sampling data, and easy integration, making it more suitable for large-scale engineering applications [5]. The basic principle is that the photodiode converts the real-time detected melt-pool light radiation signal into a voltage signal and transmits it to the computer to obtain the melt-pool light radiation signal. The melt-pool light radiation signal contains a significant amount of real-time melt-pool information, which is of significance for quality prediction and defect tracking.

Establishing the relationship between the melt pool light radiation signal and quality can help us achieve rapid results of quality during the forming process to obtain quality control measures in advance, reduce the scrap rate, avoid waste, and reduce the quality inspection cost. In this study, microhardness was selected as the quality used for modeling to predict the correlation with the melt pool radiation intensity. Because LPBF is an additive forming method with stacked melt paths and layer-by-layer accumulation, there are many factors affecting the melt pool, such as absorption ratio [6], etc. The forming process has a complex heat transfer process that affects the element distribution and microstructure characteristics of the formed sample [7,8]. This results in performance differences in microhardness between the various regions of the melt path, where the weak performance zone directly determines the service life of the formed sample [9]. For materials and components with high microhardness requirements, hardness prediction can simplify the quality control process during the forming process, reduce the scrap rate, and guarantee the forming quality with low economic and time costs. For materials and components without special hardness requirements, hardness prediction can rapidly sense the hardness distribution and indirectly assist in the development of the next heat treatment process, thus guaranteeing product quality. The future of LPBF is to achieve integrated solutions for structure, material, process, and post-treatment; therefore, the quality prediction of microhardness is vital.

Establishing a correlation between melt pool radiation intensity and quality is a key issue. Currently, the direct correspondence between the melt pool radiation intensity and forming quality is less studied, and the significant amount of forming process information contained in the melt pool radiation signal has not been fully explored and utilized. Because the LPBF forming process is complex with several influencing factors, it is challenging to conduct research and analyses based on physical models. However, the data-driven approach has the advantage of modeling complex physical problems [10], and some classical machine learning models, such as support vector machines and linear regression algorithms, have been used for the classification and prediction of forming quality in additive manufacturing [11]. An important challenge of data-driven approaches for quality prediction is the accuracy and speed of prediction. Integrated learning is a suitable solution to this problem. Compared with a single learner, integrated learning exhibits better performance and higher accuracy. The integration algorithm combines multiple weak learners to obtain a strong learning model. The principle is that different learners correct each other’s errors and improve the generalization ability and robustness of the learners to achieve ultimate accuracy improvement. Integrated learning methods are broadly classified into two categories: bagging and boosting. Bagging [12] methods are characterized by parallelization methods that do not have strong dependencies between weak learners and can be generated simultaneously. The representative algorithms are random forest algorithms. Boosting [13] methods have strong dependencies between basic learners and use serially generated random forest as a decision tree-based learner, which introduces random feature selection in the training process of decision trees and has a stronger ability to prevent overfitting. XGBoost and LightGBM are both highly scalable algorithms characterized by rapid and accurate processing of various problems and are better than traditional machine methods concerning performance, efficiency, and running speed. In this study, the three algorithms mentioned above were used for modeling.

In this study, information on the radiation intensity of the melt pool during the forming process was collected using a photodiode as a sensor; the hardness of the formed sample was predicted, and the model was interpreted using random forest, XGBoost, and LightGBM for modeling based on its process parameters and power spectrum characteristics. The model in this study is applicable to the printing equipment, printing conditions, and metal powder materials used in this experiment. Whether the same applies to cross-domain materials and equipment will be verified in a subsequent study. In Section 2, we discuss related studies on in situ monitoring and prediction models for melt pools. In Section 3, the random forest, XGBoost, and LightGBM algorithms are described, along with information on the experimental setup, melt pool intensity data pre-processing, and hardness performance testing. In Section 4, we present an analysis of the hardness results and model predictions, evaluate the performance of these machine-learning algorithms, and perform a comparative study. A summary of this study is presented in Section 5.

## 2. Related Work

### 2.1. In-Situ Monitoring of the Melt Pool

In situ monitoring of the melt pool of laser powder bed fusion is mainly performed in real-time using test devices, such as high-speed cameras, pyrometers, and photodiode sensors. Vrancken [14] et al. observed the crack generation process of tungsten metal during LPBF by in situ monitoring with high-speed cameras and analyzed the influence of process parameters (laser power, scanning speed, and beam diameter) and melt pool geometry on the cracks. Pavlov [15] et al. used a two-color pyrometer to monitor the melt pool temperature during the forming process in real-time and found that the measured values of the two-color pyrometer were very sensitive to process parameters, such as scan spacing, powder laydown thickness, and scan strategy, and they were able to detect melt pool anomalies owing to the uneven thickness of the powder bed. Coeck [16] et al. proposed a method to predict the size and location of pores by extracting and analyzing the melt pool radiation signal monitored during LPBF processing with a photodiode. These methods are effective in obtaining melt pool information during the forming process in real-time; however, the low cost of photodiodes is more suitable for industrialization. Moreover, Berumen [17] et al. combined a high-speed camera with a photodiode and found that the temperature gradient of the entire printing area could be identified by the photodiode alone; thus, the diode has significant potential for industrialization as a sensor for real-time melt pool monitoring.

### 2.2. Data-Driven Modeling Approach

Several studies have been conducted on artificial intelligence modeling based on additive manufacturing process monitoring. Francis [18] et al. used a convolutional neural network (CNN) based on the LPBF process to predict the deformation of the formed part using process parameters and thermal history as the input and deformation as the output for modeling and then compared it with the CAD model to achieve error compensation. Tapia [19] et al. applied the GP model to predict the porosity and melt-pool depth in the LPBF process and obtained promising results. Gobert [20] et al. used a high-resolution camera to collect images of each layer and used a linear support vector machine to extract and evaluate multidimensional visual features. The support vector machine was trained with the true location of defects obtained from 3D computed tomography data as labels, and the results indicated that the in situ defect monitoring accuracy was greater than 80%. Aminzadeh [21] et al. developed an online monitoring system for monitoring the fusion quality and defect formation in each layer of the LPBF process using a Bayesian classifier for training and testing. The results indicated that the system was able to detect areas with poor fusion quality or defects in each layer with true positive and negative rates of 89.5 and 82%, respectively. Duan [22] et al. performed machine learning on the time-domain features of LPBF photodiode-based melt-pool monitoring data with modeling methods that mainly support vector machine and decision tree algorithms to achieve quality predictions (density and surface roughness) and the identification of process parameters. Zhang [23] et al. extracted features such as the melt pool and spatter of different quality melt channels and used the principal component analysis algorithm for feature dimensionality reduction. The feature vectors were used as the input for modeling with a support vector machine and CNN, respectively, to classify melt lanes of different quality, and the support vector machine had an accuracy of up to 90.1%; however, the CNN had a higher classification accuracy of 92.7%.

## 3. Methodology

### 3.1. Data-Driven Predictive Modeling

(1) Random forest

**Figure 1:**
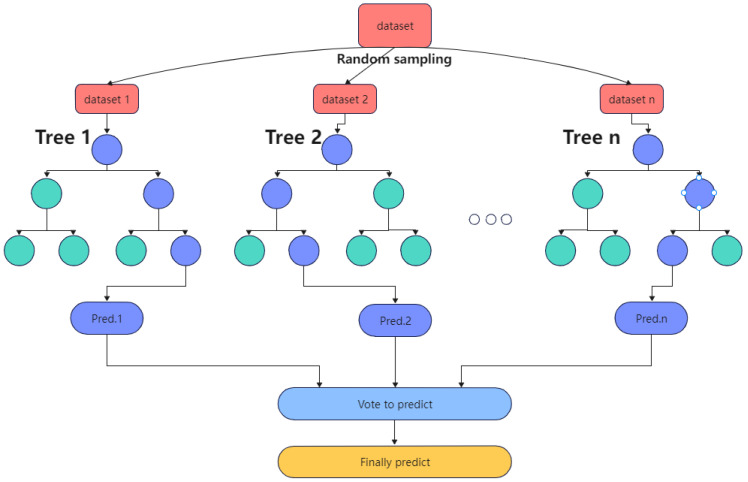
Diagram of random forest prediction flow.

Random forest is a decision tree-based bagging integrated learning method proposed by Breiman [24]. The core principle of this algorithm is to randomly select a portion of samples from the original training samples multiple times in a put-back manner to generate a new set of samples and train decision trees in each new set of samples; the generated multiple decision trees form a random forest and then vote to determine the final output by the number of votes in the decision trees. The random forest algorithm uses multiple decision trees in parallel to train the model, so each decision tree can select some samples and features, which can avoid overfitting to a certain extent. Simultaneously, each decision tree randomly selects samples and features, which enables the random forest algorithm to overcome the weak generalization ability of decision trees and improve the prediction effect of the final model. Figure 1 shows the flow of the random forest prediction model.

(2) XGBoost

**Figure 2:**
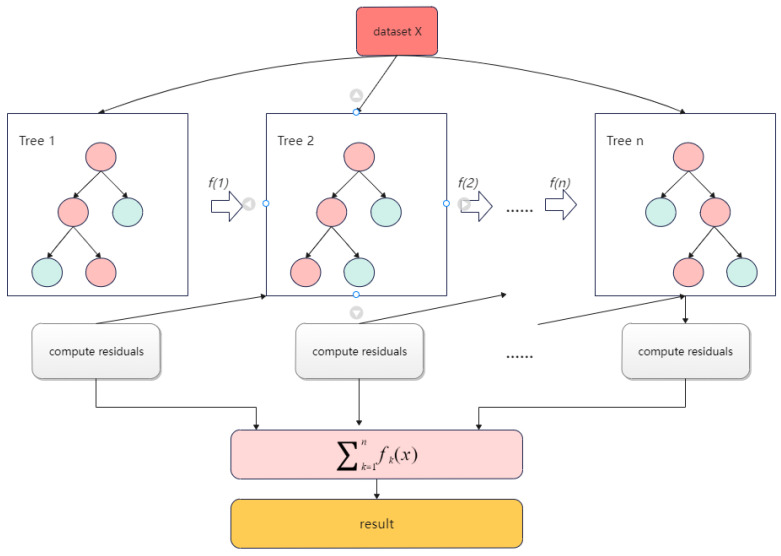
Diagram of XGBoost prediction flow.

Extreme gradient boosting (XGBoost) is a boosting learning algorithm developed by Chen at the University of Washington in 2016 [25]. The principle of XGBoost is to improve the prediction accuracy by continuously forming new decision trees and continuously performing feature splitting to fit the residuals of previous predictions to continuously decrease the residuals between the predicted and true values. XGBoost first builds a specific number of weak learners, most of which are classification regression trees, and then trains the weak learners. After training, a weighted summation was performed to obtain the final regression model. In the model-building process, iterations of new learners are added based on the residual error obtained from the previous weak learner iteration. The new learner is built on the gradient to ensure error reduction in the entire model and ultimately achieve a high-accuracy regression prediction. XGBoost introduces L1 and L2 regularization terms compared to the traditional tree model and fits the residuals with a second-order Taylor expansion of the loss function. After each iteration, XGBoost assigns the learning speed to the leaf nodes, reducing the weight of each tree and providing a better space for subsequent learning. Figure 2 shows the XGBoost prediction flow.

(3) LightGBM

**Figure 3:**
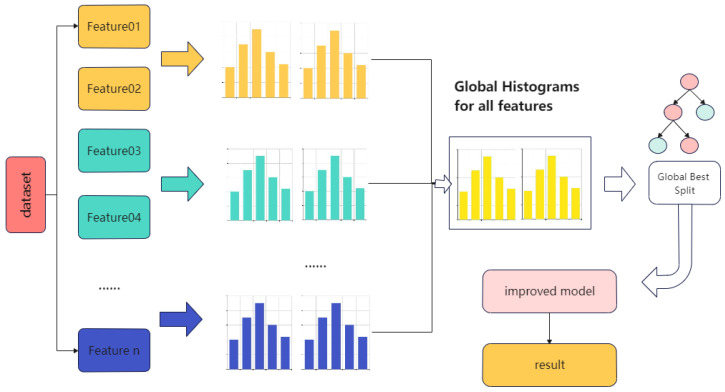
Diagram of LightGBM prediction flow.

LightGBM is a new boosting framework model proposed by Microsoft [26] that introduces gradient-based one-side sampling (GOSS) and independent feature merging techniques based on traditional GBDT, with faster training efficiency, low memory usage, higher accuracy, and support for parallelized learning, and can handle large-scale data. GOSS eliminates most of the samples with small gradients and calculates the information gain using only the remaining samples, which is a balanced algorithm to reduce data volume and ensure accuracy. Exclusive feature bundling involves bundling mutually exclusive features to reduce the dimensionality of features. The tree is constructed using the growth strategy of the leaf-wise algorithm to reduce computation. A histogram algorithm is used in LightGBM to transform the stored feature values into stored bin values to reduce memory consumption. Figure 3 shows the LightGBM prediction flow.

### 3.2. Data Collection

#### 3.2.1. Materials

The specimens in this study were prepared using a K438 high-temperature alloy powder, which is comparable in composition and properties to IN738, which is widely used internationally. In addition to excellent heat resistance and corrosion resistance, the alloy also has a medium level of high-temperature strength and good organizational stability and is widely used for long-life turbine working blades and guide blades of naval and ground industrial gas turbines working below 900 °C, as well as for turbine parts of aero engines. Its main chemical composition is shown in Table 1. The particle size distribution of the powders is shown in Table 2.

#### 3.2.2. Experimental Setup

**Figure 4:**
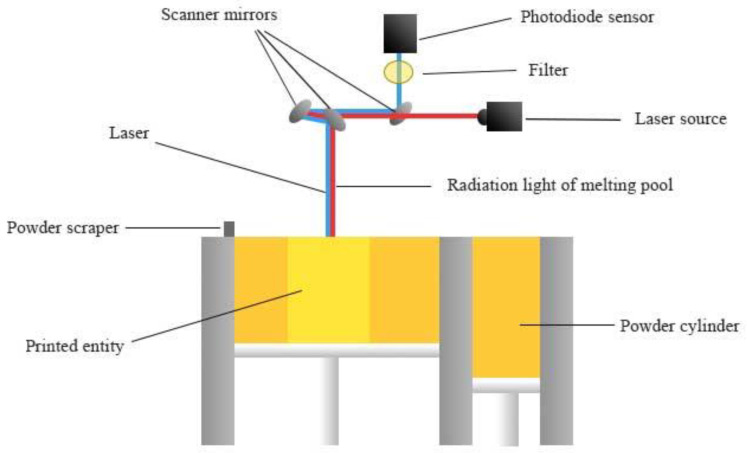
Schematic diagram of the online monitoring system of the melt pool.

The experimental specimens in this study were formed and prepared using the laser powder melting equipment of the Beijing E-plus 3D Company, which consists of a laser optical scanning system (laser optical system and scanning oscillator), working chamber, gas circulation analysis system (gas circulation system, oxygen analysis alarm system), and control system (computer, software system). The equipment is also equipped with a melt pool online monitoring system that monitors the forming process and collects melt pool radiation intensity data in real-time. Figure 4 depicts the principle of the online melt pool monitoring system. The emitted laser is reflected by the scanning mirror into the forming bin, which is partially absorbed by the powder and melted to form a melt pool. In addition to melt pool radiation in the molding chamber, there is also visible light from the headlamp and reflected laser light (1024 nm), etc. In order to avoid interference from laser-emitted light and fully reflect the characteristics of the melt pool, a bandpass filter of 750–950 nm was set. After filtering out the interfering light waves, the light in the infrared band near the peak of radiation is collected to the photodiode, which then converts the melt pool radiation signal into a voltage signal and sends it to the computer for storage, thus realizing online monitoring of the melt-pool radiation.

#### 3.2.3. Design of Experiment

(1) Forming experiments

**Figure 5:**
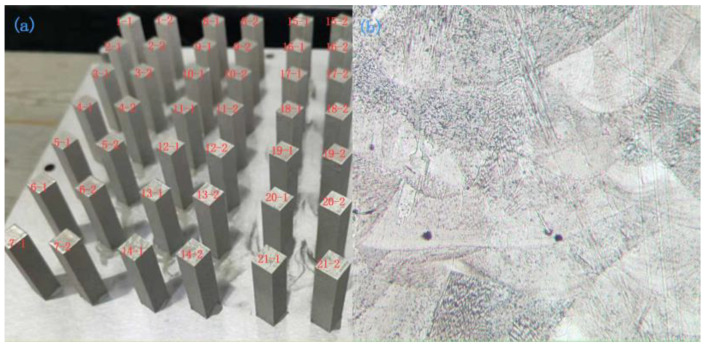
Diagram of experimental sample and microstructure. (**a**) LPBF-build plate with manufactured samples and (**b**) microstructure, XZ plane.

In order to diversify the measured hardness data and facilitate the training and prediction of the model, a total of 21 groups of specimens were printed according to different process parameters, in which the laser power of the first group varied in the range of 150–400 W, the scanning speed was 1200 mm/s, and the scanning spacing was 0.07 mm; the laser power of the second group was 270 W, the scanning spacing was 0.07 mm, and the scanning speed varied in the range of 600–2200 mm/s; the laser power of the third group is 270 W, scanning speed is 1200 mm/s, and scanning pitch is 0.02–0.15 mm. To ensure repeatability, two specimens were printed in each group, for a total of 42 specimens, each with a forming size of 10 × 10 × 40 mm^3^ and 1000 printed layers. The specific process parameters are listed in Table 3. A diagram of experimental specimens and the microstructure is shown in Figure 5.

(2) Hardness tests

In this study, we predicted the microhardness of different layers of the formed part, and the label data can be obtained by obtaining the microhardness data. Microhardness measurements were performed along the Z-axis using a microhardness tester after grinding and polishing the side surfaces of the specimens. The testing principle is to use a diamond indenter of a certain cone shape and apply a pressure generated by a mass ranging from a few grams to several hundred grams (0.2 kgf in this study) to the surface of the specimen. In this study, a 130° diamond pyramid was used as the inputting head. The diagonal length of the indentation was measured to determine the microhardness. The total length of each specimen in this study was 40 mm, with 1000 layers. We measured one microhardness value every 1 mm during the measurement, that is, one point every 25 layers; thus, 39 data points were obtained for each specimen, and a total of 1638 data points were obtained for the entire experiment.

#### 3.2.4. Data Preparation

(1) Extraction of features

Approximately 37 Mb data points were collected for the melt pool radiation intensity via the online monitoring system of the melt pool for each layer, making it difficult to obtain ideal analysis and calculation results without further processing. Our team has conducted some analyses and research on the time-domain characteristics of the LPBF melt pool radiation intensity information [19]; however, the intuitive time-domain analysis often cannot obtain the complete hidden information of melt pool radiation intensity and its comprehensive understanding. Therefore, this study will perform feature extraction and correlation processing on the melt pool radiation intensity data from the frequency-domain dimension. After obtaining the label (microhardness) data, we will find the power spectrum density for the corresponding layer of melt pool radiation intensity data and extract nine power spectrum-related features, such as power maximum, power minimum, power extreme difference, power median, mean power, center of gravity power, power root mean square, power standard deviation, and power spectrum entropy, as well as process parameter information (laser power, scanning speed, and scan spacing) and layer information. The process parameter information (laser power, scanning speed, and scan spacing) and the number of layers are also used as the input features of the prediction model, totaling 13 features, which are detailed in Table 4.

(2) Dataset partitioning

The difference in the order of magnitude between the feature data can make the objective function more dependent on attributes with large values, resulting in weak predictions; thus, the feature data are first normalized for better performance and faster convergence. We then correlated the normalized feature data to the measured hardness data individually to create the dataset used for modeling. Approximately 80% of the data was randomly selected as the training set, and 20% of the data was used as the test set. During the training process, the training data were used to tune the hyperparameters of the model while finding the best hyperparameters by grid search. The performance of the prediction model with the best hyperparameters was evaluated using the remaining test data.

## 4. Results and Discussion

### 4.1. Modeling

**Figure 6:**
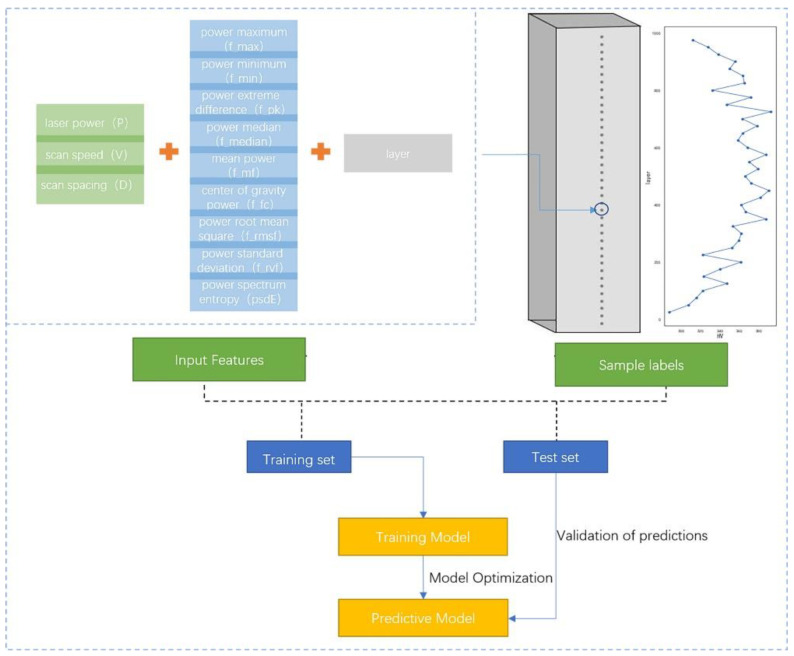
Model schematic.

**Figure 7:**
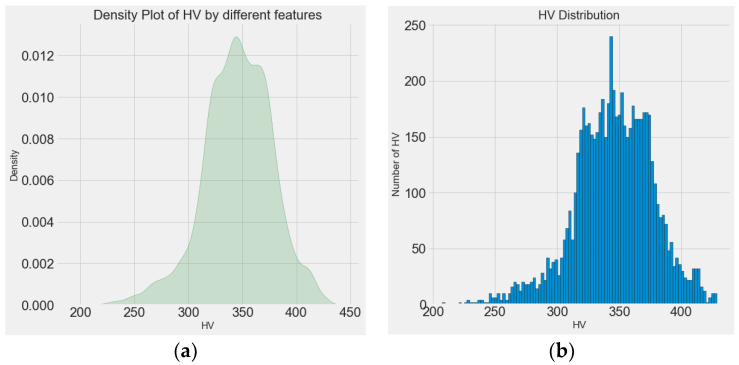
Distribution of microhardness data. (**a**) Microhardness density graph and (**b**) histogram of microhardness distribution.

Figure 6 shows a schematic diagram of the entire principle of the dataset, model, and prediction. From Figure 6, it can be observed that the 13 features consisting of a process parameter, layer, and power spectrum-based features together form one microhardness data point. Each sample was measured every 1 mm along the Z-axis (39 microhardness values were obtained), and the microhardness distribution exhibited an “arch” distribution, which is characterized by minimums at both ends and a maximum in the middle. A total of 1638 microhardness values were obtained, the distribution of which is shown in Figure 7. Figure 7a shows the density plot of the microhardness data, and Figure 7b shows the distribution histogram of the hardness data. It can be observed that the overall microhardness obtained for the entire experiment has an approximately normal distribution, and such data will have a facilitating effect on the training effect of the model.

The dataset consisting of features and labels (microhardness) was divided into training and test datasets by random sampling. The training dataset was input to the models (random forest, XGBoost, and LightGBM) for training and tuning the parameters separately to obtain the optimal parametric model. Finally, the prediction data were input to the trained models for validation, and the final prediction results were obtained.

### 4.2. Performance Evaluation

The performance evaluation is an important aspect of measuring the accuracy and precision of a model. The root mean square error (RMSE), mean absolute error (MAE), and coefficient of determination ($R^{2}$) are metrics often used to measure the accuracy of predictive models. The error metrics are defined as follows:

(1) $$RMSE=\sqrt{\sum_{i=1}^{n}\frac{{\left({\hat{y}}_{i}-y_{i}\right)}^{2}}{n}}$$

(2) $$MAE=\frac{1}{n}\sum_{i=1}^{n}|{\hat{y}}_{i}-y_{i}|$$

(3) $$R^{2}=1-\frac{\sum_{i=0}^{n}{\left({\hat{y}}_{i}-y_{i}\right)}^{2}}{\sum_{i=0}^{n}{\left(\bar{y}-y_{i}\right)}^{2}}$$

where ${\hat{y}}_{i}$ is the model predicted value, and $y_{i}$ is the true measured value.

The MAE is used to evaluate the proximity of the predicted results to the true dataset by first calculating the residuals for each data point, using the absolute value of each residual so that negative and positive residuals do not cancel out, and then taking the average of all the residuals. The RMSE, that is, the standard error, was used to measure the deviation of the predicted value from the true value. The RMSE and MAE range was [0,+∞); when the predicted value tended to the true value, the values of RMSE and MAE tended to 0, that is, the perfect model; the larger the error, the larger these values, and the less satisfactory the model effect. $R^{2}$ describes the percentage of response variation explained by the model. If $R^{2}$ is close to 100%, it indicates that the model can explain most of the variability and that the model fits the data well.

Table 5 summarizes the prediction accuracy and prediction times of XGBoost, random forest, and LightGBM. The random forest model had the best performance of RMSE, MAE, and $R^{2}$, followed by XGBoost and LightGBM. Although the performance of the random forest model is sufficient, the model trained the slowest, approximately 2.27 s, and the prediction time was 565 ms; the performance of the XGBoost model was similar compared with the random forest model. Its training time was very short, approximately 538 ms, which is 23.7% of the random forest model, and the prediction time was approximately 214 ms. LightGBM had the shortest training time, approximately 32.9 ms, which is 1.4% of the time taken by random forest, and a prediction time of 24.6 ms, which is 4.4% of the time taken by random forest. However, its model performance was poor. The overall performance of XGBoost was superior. Random forest, XGBoost, and LightGBM can adapt well and exhibit good performance even if the data set is small, as the model performance in this study is still good with the small data set, and it can be expected that the prediction accuracy can be further improved with an expansion of the data set.

### 4.3. Model Explanation

In feature selection, we ranked all features based on importance using the bagged decision tree algorithm; however, in practice, we also need to understand how these features affect the model prediction results to mine valuable information. We selected the XGBoost model with the best overall performance for model interpretation through the local interpretable model agnostic explanation [27] algorithm.

**Figure 8:**
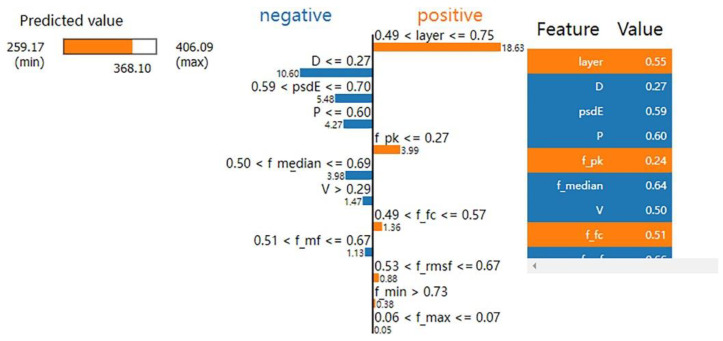
Infographic of data X features influencing weights.

A randomly selected hardness value is denoted as data X for the analysis, and Figure 8 depicts the influence and weight of each feature of data X on the predicted value. The true value of this microhardness was 368.10, and the predicted value was 368.0988, which is an accurate result. The left part of Figure 8 shows the prediction range, and it is evident from the middle part that features such as the layer number indicator, power polar difference, power maximum, and root mean square power in the prediction of the microhardness value at this point make positive contributions to the model prediction results. Features such as scan spacing and median power have negative contributions, and the layer number indicator has the most significant influence on the model prediction value when the layer number indicator is between 0.49 and 0.75. The main reason for the significant contribution of the number of layers to the model is that laser powder bed melting is a layer-by-layer stacking process, and because of the existence of heat accumulation, the temperature gradients of different layers are different, and the final effect on hardness is also different; therefore, the number of layers is also a vital feature. The process parameters contribute significantly to the model in this data model prediction, the scan spacing being the most significant, followed by the laser power and scan speed. Based on the power spectrum features in the power spectrum entropy, the median power of the model is an indispensable and vital feature. The right side indicates the actual index value of each feature of the data.

## 5. Conclusions

The main objective of this study was to use a data-driven approach to predict the microhardness of LPBF samples and to obtain the microhardness values of future molded samples directly during the melting process. Not only can the process adjustments be made in time if production requirements are not met, reducing scrap and waste, but the microhardness distribution can also be rapidly determined without the need for experiments, providing guidance for the next heat treatment process. The main work of this paper is based on a data-driven approach to predicting microhardness, and the main conclusions are as follows:

(1) We collected the melt pool radiation intensity data of the K438 powder printing process and processed this data to obtain nine features based on the power spectrum, and then formed the input features together with the process parameter information (laser power, scanning speed, scanning spacing) and layer information.
(2) Hardness measurements are performed along the Z-axis direction, and the obtained hardness data are corresponded to the melt pool radiation intensity characteristics data at the corresponding locations to form a data set, while 80% are randomly selected as the training set and the remaining 20% as the test set.
(3) We selected random forest, XGBoost, and LightGBM for the training and prediction and evaluated their performances individually. The results indicate that all three algorithms, random forest, XGBoost, and LightGBM can predict the microhardness of the samples with high accuracy, with random forest performing the best, followed by XGBoost and LightGBM. However, based on training and prediction time, LightGBM performed the best, followed by XGBoost and random forest. The best overall performer was XGBoost, which had a very short prediction time while still ensuring accuracy and excellent performance for subsequent use in industry.

This study was based on one type of material and equipment for analysis and modeling and achieved satisfactory prediction results; however, if different materials and equipment were to be used, the prediction accuracy of the model would need to be investigated. After considering these problems, based on our existing experience and foundation, we propose that it is necessary to establish a metal additive database and further study the universal prediction model based on additive large data, which is more applicable to mass production. We have started investigating the metal additive database construction, and it is foreseeable that with the analysis and processing of massive data volumes, the generation of universal models will make significant progress in solving the consistency problem of additive manufacturing.

## Figures and Tables

**Table 1:** Chemical composition of K438.

Element	Cr	Co	W	Mo	Al	Ti	Nb	Ta	Si	P	C	Ni
wt.%	15.90	8.28	2.71	1.56	3.53	3.05	0.94	1.83	0.20	0.41	0.19	61.30

**Table 2:** Powder particle distribution of K438.

Powder Particle Distribution/(μm)			Hall Flow Rate/(s/50 g)
D10	D50	D90	20.72
16.85	35.19	56.29	

**Table 3:** Process parameters.

Specimen Number	Laser Power/ W	Scanning Speed/ (mm/s)	Spacing Interval/ mm	Powder Layer Thickness/μm
1	150	1200	0.07	40
2	200			
3	250			
4	270			
5	290			
6	350			
7	400			
8	270	600	0.07	
9		800		
10		1000		
11		1200		
12		1500		
13		1800		
14		2200		
15	270	1200	0.02	
16			0.04	
17			0.06	
18			0.07	
19			0.08	
20			0.1	
21			0.15	

**Table 4:** Feature details.

Feature Type	Feature Name	Formula	Meaning
Features based on power spectrum	power maximum(f_max)	/	All are features that simply describe the dispersion information of power.
	power minimum(f_min)	/	
	power extremedifference (f_pk)		
	power median(f_median)	/	This feature measures the energy concentration tendency of the power spectrum.
	mean power(f_mf)	$f\_mf=\frac{1}{N}\sum_{n=1}^{N}u(n)$	This feature is the arithmetic average of the power spectrum energy and is susceptible to extreme values.
	center of gravitypower (f_fc)	$f\_fc=\frac{\sum_{n=1}^{N}f(n)u(n)}{\sum_{n=1}^{N}u(n)}$	This feature describes the frequency of the signal component with large components in the power spectrum and reflects the distribution of the signal power spectrum.
	power root meansquare (f_rmsf)	$f\_rmsf=\sqrt{\frac{\sum_{n=1}^{N}f^{2}(n)u(n)}{\sum_{n=1}^{N}u(n)}}$	This feature is the weighted average of the signal power squared followed by the arithmetic square root.
	power standarddeviation (f_rvf)	$f\_rvf=\sqrt{\frac{\sum_{n=1}^{N}{\left(f(n)-f\_fc\right)}^{2}u(n)}{\sum_{n=1}^{N}u(n)}}$	This feature describes the energy dispersion degree of the power spectrum with the inertia radius centered on the gravity center frequency.
	power spectrumentropy (psdE)	/	Based on information entropy, this feature describes the spectral structure of the power spectrum and describes the uncertainty and complexity of the signal power spectrum.
Features based on process parameters	laser power (P)	/	/
	scan speed (V)	/	/
	scan spacing (D)	/	/
Layer feature	layer	/	/

Note: u(n) (*n* = 1, 2…, *N*) denotes the power spectral density amplitude, *N* the number of spectral lines, f(n) the frequency of the *n*-th spectral line, and $\bar{u}(n)$ the average value of the power spectral density amplitude.

**Table 5:** Model performance evaluation.

Model	RMSE	MAE	$R^{2}$	Training Time	Predicted Time
Random Forest	9.2867	5.9821	0.9141	2.27 s ± 1.1 ms	565 ms ± 3.36 ms
XGBoost	10.2082	7.2856	0.8962	538 ms ± 7.49 ms	214 ms ± 2.8 ms
Lightgbm	15.2875	11.0385	0.844	32.9 ms ± 1.87 ms	24.6 ms ± 1.85 ms

## Data Availability

Not applicable.
